# Vaccine Impact Data Should Support Country Decision Making

**DOI:** 10.1093/infdis/jix187

**Published:** 2017-04-18

**Authors:** E Anthony S Nelson, A Duncan Steele

**Affiliations:** 1 Department of Paediatrics, Chinese University of Hong Kong, Hong Kong Special Administrative Region, People’s Republic of China; 2 Enteric and Diarrheal Diseases, Bill & Melinda Gates Foundation, Seattle, Washington

**Keywords:** Rotavirus vaccine, rotavirus, diarrhea, vaccine impact, NITAG

**(See the major article by Burnett et al on pages 1666–72.)**

By December 2016, 81 of the 194 World Health Organization (WHO) member states (42%) had introduced rotavirus vaccine into their National Immunization Program (NIP); and several others had introduced it at the subnational level. The promising early introduction of rotavirus vaccines in 8 countries in 2006, when the vaccines were first licensed in Europe (Rotarix, GSK, Belgium) and the United States (RotaTeq, Merck), included both Gavi (a global vaccine initiative)–eligible and high-income countries. Prequalification of the 2 vaccines in 2008 was accompanied by a WHO policy recommendation in 2009 that all countries should include rotavirus vaccines in their NIPs, and particularly those with high burden of diarrheal illness [1], thus opening the door to rapid global scale-up.

Encouragingly, and despite the modest efficacy observed in low-income countries (LICs) and lower- to middle-income countries (LMICs) in Africa and Asia [2–4], there was a surge of national introductions, mostly with Gavi funding support between 2012 and 2014. However, introductions have slowed down in recent years despite Gavi funding, and never started in earnest in Asia, a region with large-birth-cohort countries carrying substantial disease.

The review by Burnett and colleagues in this issue of *The Journal of Infectious Diseases*, demonstrating the global impact of rotavirus vaccines on rates of acute gastroenteritis (AGE) mortality and hospitalization in children <5 years of age, emphasizes that the decision to introduce rotavirus vaccines was correct. The impact of these vaccines on rotavirus-related hospitalizations and the proportion of rotavirus-associated diarrhea admissions were evaluated before and after introduction of the vaccines. Data were available from 27 countries that have introduced rotavirus vaccine into their NIPs, covering the 10-year period from licensure (2006) to December 2016. This review complements and augments data from meta-analyses of postintroduction vaccine effectiveness studies conducted previously [5, 6].

Mortality from AGE in children <5 years of age in countries in the high (n = 3) and medium (n = 11) mortality strata fell 36% and 50%, respectively, and 42% overall (range, 3%–64%). In infants, the overall reduction was 31%. Although these ecological data cannot prove causality, these reductions in AGE mortality declines have been coincident with rotavirus vaccine introductions in a range of countries. The fact that some high- and medium-mortality countries have yet to introduce vaccine is a cause for concern. National immunization technical advisory groups (NITAGs) and decision makers in Gavi-eligible nonadopter countries should carefully review their decisions not to introduce these life-saving vaccines, in the face of these data. Introduction decisions are understandably harder for the non-Gavi-eligible LMICs where AGE mortality is lower and vaccine price may be anticipated to be high and less affordable.

Decisions to introduce a new vaccine into NIPs are influenced by many factors including local disease burden, vaccine efficacy and safety, and cost-effectiveness of the vaccines. Rotavirus disease burden has been recognized for decades, and regional surveillance networks have informed countries about the ubiquitous nature and high mortality in infants and young children <5 years of age associated with the disease [7]. High-income and upper- to middle-income countries make decisions influenced by cost-effectiveness analyses based on strong efficacy against rotavirus hospitalizations. For LMICs and LICs in Africa and Asia, efficacy data were modest (45%–65%) [1–4]. Despite this, increasing national introductions in sub-Saharan Africa have occurred since the first Gavi-eligible country introductions in 2012 [8]. By December 2016, 28 African countries have implemented rotavirus vaccines. However, in this same period, only 2 subnational introductions occurred in Asia (ie, Thailand with a pilot program and the Philippines).

The lack of rotavirus vaccine implementation in Asia has now turned an important corner. India introduced an indigenously developed vaccine [9] in March 2016 in 4 states representing approximately 9% of the birth cohort, and was scaled up in 5 additional states covering approximately 50% of the birth cohort in 2017. This represents an acceleration for new vaccine introductions in India and is welcome because of the high rotavirus mortality there [10]. Pakistan introduced rotavirus vaccine, with Gavi financing, in several districts in Punjab in January 2017, with plans for national scale-up. Finally, Bangladesh has been approved for Gavi-supported introduction in 2018.

In addition, 2 large African countries with high rotavirus mortality are approved for Gavi funding to introduce the vaccines in 2018 (Nigeria and Democratic Republic of Congo). The dramatic impact of rotavirus vaccines on rotavirus-associated hospitalizations and deaths described by Burnett et al support the decisions by these large countries with high rotavirus mortality to introduce rotavirus vaccines, and will lead to greater global health impacts.

However, other global factors are crucial to widespread introduction including pricing of the vaccine for the market, and sufficient global supply for countries [11]. There is also promise on this front. Two new rotavirus vaccines have been licensed by the Drugs Controller General of India after large phase 3 studies [12, 13]. One vaccine, Rotavac, a monovalent human rotavirus strain developed by Bharat Biotech International Ltd, Hyderabad, is being progressively rolled out in India, as described above, with embedded programmatic and safety monitoring and vaccine effectiveness evaluations in progress. The vaccine has been submitted for WHO prequalification, which would increase global vaccine supply of a vaccine that was publicly offered to the Gavi market at approximately US$3/course [9]. The second, Rotasiil, a pentavalent bovine-human reassortant vaccine developed by Serum Institute of India, Pune, has completed a phase 3 efficacy study in India which has yet to be published although the results are pending, and a similar study has been conducted in Niger by Médecins Sans Frontières [13]. The manufacturer has also submitted the vaccine for WHO prequalification, and the price of the vaccine is estimated to fall between the Gavi prices for the 2 existing WHO-prequalified vaccines, Rotarix and RotaTeq [14]. Thus, there is the potential for additional WHO-prequalified, lower-priced vaccines for country uptake.

Countries considering introduction now will increasingly have additional data on vaccine safety and effectiveness. Burnett and colleagues’ review will likely provide important information to these NITAGs and other decision-making bodies. This review will also be important for the earlier-adopter countries by way of validating their earlier introduction decisions. Finally, the information will also be of value to Gavi, the United Nations Children’s Fund (UNICEF), and other international bodies tasked with providing resources and support for rotavirus vaccine introduction in LICs and LMICs.

Could concerns about the association of rotavirus vaccines with intussusception be a factor in the decision not to introduce these vaccines? This seems unlikely, as several authoritative bodies, including WHO and the Global Advisory Committee for Vaccine Safety, have strongly recommended that the benefits of vaccination far outweigh the very small risk of intussusception. Therefore, we can assume that a decision not to introduce an otherwise safe and highly effective vaccine for a ubiquitous disease that causes significant mortality in LICs and high morbidity in all countries is due to other factors, and cost of vaccine, or perceptions of cost of vaccine, is a likely suspect [8]. Once prequalified by WHO, the new rotavirus vaccines could help address this issue.

A McKinsey & Co network analysis mapped the complex relationships among the influencers of the decision-making process for new vaccine introduction in 4 countries (Egypt, Mauritania, Mexico, Zambia) [15]. Although international organizations played an important role in the vaccine introduction process, the analysis showed that countries did not seek the advice of global experts or share information and experiences from other countries. Involving ministries of finance at an early stage in the decision-making process was recommended, highlighting the key role of vaccine cost. Although industries have indicated willingness to provide tiered pricing [16], vaccine prices, outside the Gavi mechanism, are opaque and highly variable. In an attempt to improve price transparency, WHO asks member states to anonymously report vaccine prices through its vaccine product, price, and procurement (V3P) reporting mechanism [17]. This database shows that some high-income countries pay as little as US$33 per rotavirus vaccine course, whereas others pay US$122. Prices for upper- to middle-income countries vary from US$13 to US$22 per course.

The review by Burnett and colleagues will bolster the evidence base, enabling all countries to consider following WHO’s 2013 recommendation for universal rotavirus vaccine introduction [18]. However, it is likely that these data on impact of rotavirus vaccines on disease burden, although necessary, are not sufficient. Countries of all income levels need transparent mechanisms to reassure their decision makers that rotavirus vaccine can be purchased at prices that are both fair and sustainable.

## Notes

***Potential conflicts of interest.*** Both authors: No reported conflicts of interest. Both authors have submitted the ICMJE Form for Disclosure of Potential Conflicts of Interest. Conflicts that the editors consider relevant to the content of the manuscript have been disclosed.

## References

[1] World Health Organization. Rotavirus vaccination. Wkly Epidemiol Rec 2009; 84:232–6.

[2] Madhi SA Cunliffe NA Steele D Effect of human rotavirus vaccine on severe diarrhea in African infants. N Engl J Med 2010; 362:289–98. 20107214 10.1056/NEJMoa0904797

[3] Armah GE Sow SO Breiman RF Efficacy of pentavalent rotavirus vaccine against severe rotavirus gastroenteritis in infants in developing countries in sub-Saharan Africa: a randomised, double-blind, placebo-controlled trial. Lancet 2010; 376:606–14. 20692030 10.1016/S0140-6736(10)60889-6

[4] Zaman K Dang DA Victor JC Efficacy of pentavalent rotavirus vaccine against severe rotavirus gastroenteritis in infants in developing countries in Asia: a randomised, double-blind, placebo-controlled trial. Lancet 2010; 376:615–23. 20692031 10.1016/S0140-6736(10)60755-6

[5] Santos VS Marques DP Martins-Filho PR Cuevas LE Gurgel RQ Effectiveness of rotavirus vaccines against rotavirus infection and hospitalization in Latin America: systematic review and meta-analysis. Infect Dis Poverty 2016; 5:83. 27514855 10.1186/s40249-016-0173-2 PMC4982225

[6] Lamberti LM Ashraf S Walker CL Black RE A systematic review of the effect of rotavirus vaccination on diarrhea outcomes among children younger than 5 years. Pediatr Infect Dis J 2016; 35:992–8. 27254030 10.1097/INF.0000000000001232

[7] Agocs MM Serhan F Yen C WHO global rotavirus surveillance network: a strategic review of the first 5 years, 2008–2012. MMWR Morb Mortal Wkly Rep 2014; 63:634–7. 25055187 PMC5779425

[8] Nelson EA de Quadros CA Santosham M Parashar UD Steele D Overcoming perceptions of financial barriers to rotavirus vaccine introduction in Asia. Hum Vaccin Immunother 2013; 9:2418–26. 23955246 10.4161/hv.26107 PMC3981852

[9] Bhan MK Glass RI Ella KM Team science and the creation of a novel rotavirus vaccine in India: a new framework for vaccine development. Lancet 2014; 383:2180–3. 24629993 10.1016/S0140-6736(14)60191-4

[10] Kang G Kelkar SD Chitambar SD Ray P Naik T Epidemiological profile of rotaviral infection in India: challenges for the 21st century. J Infect Dis 2005; 192(suppl 1):S120–6. 16088795 10.1086/431496 PMC2464020

[11] Makinen M Kaddar M Molldrem V Wilson L New vaccine adoption in lower-middle-income countries. Health Policy Plan 2012; 27(suppl 2): ii39–49. 22513731 10.1093/heapol/czs036

[12] Bhandari N Rongsen-Chandola T Bavdekar A; India Rotavirus Vaccine Group Efficacy of a monovalent human-bovine (116E) rotavirus vaccine in Indian infants: a randomised, double-blind, placebo-controlled trial. Lancet 2014; 383:2136–43. 24629994 10.1016/S0140-6736(13)62630-6 PMC4532697

[13] Isanaka S Guindo O Langendorf C Efficacy of a low-cost, heat-stable oral rotavirus vaccine in Niger. N Engl J Med 2017; 376:1121–30. 28328346 10.1056/NEJMoa1609462

[14] United Nations Children’s Fund (UNICEF). Rotavirus vaccine: supply and demand update. https://www unicef org/supply/files/Rotavirus_Vaccine_Supply_and_Demand_Update_-_October_2016 pdf 2016 Oct 20 Accessed 13 February 2017.

[15] McKinsey & Co. Mapping influencers in the vaccine introduction decision-making process in developing countries. http://www who int/immunization/stakeholders/mapping_vaccine_decision_making_networks pdf 2007 Mar 22 Accessed 9 July 2013.

[16] Stéphenne J Vaccines as a global imperative—a business perspective. Health Aff (Millwood) 2011; 30:1042–8. 21653955 10.1377/hlthaff.2011.0338

[17] World Health Organization. Vaccine product, price and procurement (V3P) web platform. http://www who int/immunization/programmes_systems/procurement/v3p/platform/en/ 2017 Mar 1 Accessed 1 March 2017.

[18] World Health Organization. Rotavirus vaccines: WHO position paper—January 2013. Wkly Epidemiol Rec 2013; 88:49–64. 23424730

