# Erratum

**DOI:** 10.1093/infdis/jiy077

**Published:** 2018-03-23

“Vaccine Impact Data Should Support Country Decision Making” by Nelson and Steele [J Infect Dis 2017; 215(11): 1634–6]. This is an Open Access article and should have contained the following copyright line: © The Author(s) 2018. Published by Oxford University Press for the Infectious Diseases Society of America. This is an Open Access article distributed under the terms of the Creative Commons Attribution License (http://creativecommons.org/licenses/by/4.0/), which permits unrestricted reuse, distribution, and reproduction in any medium, provided the original work is properly cited.

The authors regret the error.

